# Introduction to the Special Issue of *IJERPH* Entitled “Prenatal Exposure to Environmental Pollutants and Other Stressors: Impacts on Fetal Development, Birth Outcomes, Children’s Health and Beyond”

**DOI:** 10.3390/ijerph19148816

**Published:** 2022-07-20

**Authors:** Halina B. Röllin

**Affiliations:** Faculty of Health Sciences, School of Health Systems and Public Health, University of Pretoria, Pretoria 0001, South Africa; halina.rollin@up.ac.za

## 1. Introduction

Environmental pollution is a major cause of global diseases, death and disability, with a toll greater than that caused by communicable diseases, such as HIV/AIDS, malaria and tuberculosis, combined. More than nine million deaths globally are attributed to environmental pollution annually, with 94% of pollution-related deaths occurring in low-income and lower middle-income countries [1].

The link between environmental pollutants and adverse reproductive and developmental health outcomes is well established. Of major concern are the effects of environmental pollutants on the developing fetus, early life stages and into adulthood. Long-term subtle effects of sustained exposure to pollutants can affect reproductive health and pregnancy outcomes, reduce defenses against diseases, affect children’s mental development and increase the risk of cancer [2].

The reduction in early life exposures to pollutants is a priority as these exposures pose a threat to fetal development, starting prior to pre-conception and continuing across all stages of pregnancy. In addition, it has been shown that exposure to pollutants in the first one thousand days of life may impair children’s health and disrupt developmental processes, resulting in stunted growth and damage to vital organs, such as the brain, lungs, and reproductive and immune systems, among other effects [3].

## 2. Short Overview of Prenatal Exposure to Toxic Environmental Pollutants and Potential Health Impacts

A major pollution driver, globally, is the use of biomass fuels, such as wood, charcoal, crop residues for cooking and heating, by 40% of the population. The combustion of these fuels produces complex and toxic mixtures of gases, fine particulate matter, volatile organic compounds and toxic metals [4]. Air pollution in households is a major contributor to the global burden of disease, with seven million premature deaths annually linked to air pollution alone [5]. As a result, vulnerable populations, such as women of childbearing age and children, are exposed to air particulate matter and gases in concentrations that far exceed safety standards. 

Many studies have reported that air pollution exposure may result in fetal thrombosis, an accumulation of arsenic in maternal and cord blood, preterm delivery, low birth weight, congenital heart defects and other impacts [6,7,8]. It is also reported that the timing of pollutant exposures during pregnancy may produce different effects (e.g., exposure during the second month of pregnancy may produce certain heart defects, and exposure during last trimester may affect fetal birth weight) [9]. Furthermore, it has been reported that exposure to fine air particulates during pregnancy can negatively affect fetus development, especially injuring the brain, and increasing the risk of premature birth or low birth weight. Exposure during infancy and early childhood stages may affect the lungs resulting in the potential development of asthma, pneumonia and chronic pulmonary disease [10,11]. These findings are of particular concern and are well-aligned with WHO reports that more than 90% of children are breathing toxic air daily [12].

Metals and other elements are environmental pollutants that are of natural or anthropogenic origin. Although some metals are needed to sustain life (e.g., for enzyme reactions, energy metabolism and cellular activities), all metals can be toxic if present in high enough concentrations. Metals occur in different forms (ions, vapors, salts) with some forms being more toxic than others, necessitating speciation of these forms when studying their toxic effects. It is known that the toxicity of metals released even in minute concentrations into the environment exceeds that of all other radioactive and organic pollutants, combined [13]. Major risk factors for exposure to metals include pregnancy, age, health status, nutrition, housing, and socio-economic status. Age is one of the most important risk factors in the case of infants and young children because of their body size, combined with the relatively higher rates of uptake of toxins from the lungs and gastro-intestinal tract, and the immaturity of the blood–brain barrier. The effects of exposures to toxic metals, such as lead, mercury, manganese and cadmium, among others, are of concern due to their well-established role in pre- and post-natal toxicity [13]. It has been shown that there is no safe level of exposure to lead, particularly in relation to the developing central nervous system in the fetus and early life stages; furthermore, exposure to lead in utero is associated with pre-term birth, stillbirths and neonatal morbidity and mortality [14,15]. Of particular concern is the mobilization of lead stored in bones (a major target tissue for lead storage) during physiological and pathological conditions and events, such as pregnancy, lactation, osteoporosis and renal disease [16,17]. Furthermore, there is a documented relationship between childhood lead exposure and the emergence of aggressive behavior and early juvenile delinquency and subsequent adult criminality in later life [18,19]. 

Another toxic metal of concern is mercury that occurs naturally as elemental mercury and as organic and inorganic compounds. The anthropogenic sources of mercury are mining, combustion of fossil fuels, particularly coal, waste incineration, the chlorine–alkali industry, non-ferrous metal production and paper manufacture, use of mercury in thermometers, barometers, dental fillings, batteries and fluorescent lamps. Another source of mercury exposure is informal rudimentary gold mining activities, which continue to increase in most, if not all, developing countries. Environmental and health impacts resulting from the misuse of mercury and the impacts of mercury on international water bodies continue to be a global concern [20,21]. Mercury is a nerve toxin and, therefore, the main health concern is its effect on the brain, particularly in the growing fetus and young children. Exposure to methyl mercury has been recognized as a cause of neurological disturbances and death in human adults, and this organic form of mercury is known to produce teratogenic anomalies in neonates [22,23]. 

Manganese, another metal of concern, is widely used in industry and agriculture. Fungicides and fertilizers containing manganese are still extensively used, particularly in developing countries, as are fossil fuels, another significant source of manganese. The gasoline additive methylcyclopentadienyl manganese tricarbonyl (MMT) is a manganese compound that was introduced to automobile fuel formulas as an octane boosting and anti-knock agent, to replace or reduce the lead content in petrol in some countries, because of the well-established toxicity of lead. This substitution has not been entirely successful given that MMT also has toxic effects [24]. Biologically, manganese is an essential metal needed for mitochondrial oxidative processes for all living mammals, including humans [25]. Both, the deficiency and excess of manganese may have detrimental health effects. Importantly, excessive intake of manganese, either through inhalation or ingestion, may result in pathology, particularly to the central nervous system (CNS) [26]. Infants have an increased absorption and retention capacity for ingested manganese when compared to adults. Their under-developed blood–brain barrier may allow for relatively free ingress of manganese into the brain [27]. It has been reported that neonates may be particularly vulnerable and the elderly may also be at risk from excessive exposure to manganese, due to the increased susceptibility of aging brain cells to injury [28]. 

The interaction of toxic metals and other elements with essential trace elements, such as zinc, copper, iron and selenium, in a living organism following environmental or occupational exposure plays an integral role in their toxicity. These elements are also crucial for reproductive processes [29]. The toxicity of elements can be increased or lessened by the metabolic status of essential trace elements; the latter can be affected either through inborn genetic defects, exposure to toxic substances or diet. An example of this interaction is chronic low-level exposure to lead during pregnancy and early childhood development. In the presence of stable concentrations of essential trace elements, exposures to even low levels of lead (and not overt toxicity) can result in behavioral impacts [30]. 

Toxic persistent organic pollutants (POPs) are another area of concern. POPs include man-made synthetic chemicals that are highly resistant to biodegradation with a high affinity for bioaccumulation (due to their hydrophobic and/or lipophilic nature) and biomagnification in the environment and living organisms, including humans. Once deposited in adipose tissue, they form a stable compound that becomes a lasting toxic burden to the body [31]. Populations are exposed to mixtures of POPs originating either from local and/or geographically far removed sources [32]. Studies on health effects in humans show similarity to those in animals, affecting neuro development, thyroid and immune function [33,34,35,36]. POPs and their compounds are known to influence reproductive health and pregnancy outcomes, reduce defense against diseases, affect children’s physical and mental development, and/or increase the risk of cancer [37,38,39]. POPs are also transferred from mother to child during pregnancy and through breastfeeding. Although breast milk is the best source of nutrition for infants and provides a range of benefits for growth immunity and development, toxic chemicals, such as polychlorinated biphenyls (PCBs), DDT (dichloro-diphenyl-trichloroethane) and its metabolites, dioxins, dibenzofurans and heavy metals, are often found in breast milk [40,41]. Another concern is that POPs compounds have the ability to exert negative health effects on humans. These detrimental effects are often subtle, long-term, sometimes trans-generational, and difficult to measure even in long-term epidemiological studies in large populations. 

Nano- and microplastics also belong to the organic pollutants and their toxic effects on pregnancy, placental transfer, birth outcomes and early life are not yet fully understood [42]. Recent research suggests that microplastics and their additives can deposit in organs, penetrate cell membranes and cross the placental barrier in exposed cells or laboratory animals [43]. 

Environmental pollutants contain a number of toxic substances that are detrimental to the health of the global population, with pregnant women and their unborn children being most affected. When studying the early life exposure to toxic substances, one needs to understand the role of placenta, amniotic fluid and umbilical cord involvement in this process, as these biological structures are responsible for the growth and development of the fetus during pregnancy and regulate the absorption of nutrients and the elimination of waste products [44]. If these functions are interrupted by toxic substances, the detoxifying process will be altered, affecting the transport of some of the essential elements responsible for the development of the fetus [45,46]. 

It is also paramount to study the synergistic interactions of environmental toxins. Research into the possible synergistic interaction of low doses of different toxic substances, particularly in women of childbearing age, infants and young children, should be a priority area of investigation. Human studies on two different cohorts of children in the Faroe Islands and in the Seychelles (both have island populations that consume fish contaminated with methyl mercury) showed different levels of neuropsychological defects. These defects were found to be attributed to the synergistic interaction of PCBs found in the mothers’ dietary consumption of whale meat and blubber in the Faroe Islands population [47,48]. Additive and synergistic effects of low levels of organochlorine pesticides were found to cause significantly greater proliferation of tumor cells than exposures to individual toxic substances [49,50]. The synergistic effects of combinations of different pesticides have been found to be much more than simply additive; they are exponential. Combinations of endosulfan, dieldrin, toxaphene and chlordane produce estrogenic effects 500 to 1000 times higher than the individual effects of each substance [51,52]. The studies showed that very high concentrations of endosulfan (160 times) and dieldrin (1600 times), as single compounds, had to be used to match the estrogenicity caused by a combination of the two compounds. Chlordane, which exhibits no estrogenic activity on its own, proved almost as potent as dieldrin or toxaphene in boosting the estrogenicity when combined with endosulfan [53]. The synergistic effects may also be responsible for the fact that the distribution of toxic waste dump sites parallels closely the sites of the highest breast cancer mortality [54]. 

## 3. Conclusions

There is growing concern for the future of the global environment and the United Nations Conference on the Environment and Development (UNCED) held in Rio de Janeiro in 1992 recognized the link between healthy people and healthy environments [55] as a prerequisite for meeting the United Nations’ Sustainable Development Goals (UN SDGs 2030).

The aim of this Special Issue of *IJERPH* entitled ‘Prenatal Exposure to Environmental Pollutants and Other Stressors: Impacts on Fetal Development, Birth Outcomes, Children’s Health and Beyond’ is to offer a platform for researchers to share their research findings, as well as scientific interpretations and opinions pertaining to prenatal exposures to environmental pollutants and other stressors that may impact not only on birth outcomes and children’s development, but also on morbidity and mortality in adulthood. 

Another aim is to bring together researchers from different countries and continents to share their expertise and advance our understanding of the complex interaction between exposure to contaminants during the prenatal stages, birth outcomes and health risks in children. Multidisciplinary research is needed in this rapidly evolving field to understand the diversity of exposures from a global perspective and the associated health effects. Moreover, it will be important to link recent research findings with the achieving of the objectives of the UN SDGs 2030.

Although reproductive health is an ongoing priority worldwide, living conditions and access to health care are defined by the geography, climatic conditions and economic structures of individual countries. Early life exposures are also affected by health and nutritional status, as well as the lifestyle of pregnant women; these combined factors are widely recognized to influence birth outcomes and children’s health into adulthood. 

Original research articles and reviews are welcome. Research areas may include (but are not limited to) the following: prenatal exposures to environmental pollutants; birth outcomes; the longitudinal studies of birth cohorts; sex/gender aspects in response to pollutant exposures; the impact of specific exposures on long-term health effects; disease susceptibility associated with genetic, epigenetic and lifestyle factors; nutritional effects; climatic influences; methodological and epidemiological studies; toxicological studies; the potential impacts of pre-natal exposures on the development of non-communicable diseases in neonates and young children; and issues that inform prevention strategies, policy formulation, and the interface and overlap between environmental and public health. 

*IJERPH* and I look forward to receiving your contributions and to creating impactful collaborations across different areas of expertise and geographical regions.
